# The Effect of Engagement in Everyday Occupations, Role Overload and Social Support on Health and Life Satisfaction among Mothers

**DOI:** 10.3390/ijerph120606045

**Published:** 2015-05-28

**Authors:** Michal Avrech Bar, Tal Jarus

**Affiliations:** 1 Department of Occupational Therapy, School of Health Professions, Sackler Faculty of Medicine, Tel Aviv University, Tel Aviv 69978, Israel; 2 Department of Occupational Science and Occupational Therapy, Faculty of Medicine, University of British Columbia, T325 Koerner Pavilion, 2211 Wesbrook Mall, Vancouver, BC V6T 2B5, Canada; E-Mail: tal.jarus@ubc.ca

**Keywords:** mothering, occupations, mental health, physical health, life satisfaction, Structural Equation Modeling

## Abstract

One of the founding assumptions underlying the health professions is the belief that there is a strong relationship between engagement in occupations, health, and wellbeing. The ability to perform everyday occupations (occupational performance) has a positive effect on health and wellbeing. However, there is also conflicting evidence indicating that participation in multiple roles or in certain occupations may lead to poorer health. Therefore, there is a need to better understand this relationship. The purpose of the present study was to examine three possible theoretical models to explain mothers’ health and life satisfaction from the perspective of their occupational performance, their role load, and their social support. 150 married mothers, ages of 25–45, who had at least one child between the ages of one to ten years, participated in the study. Data were collected by using seven self-report questionnaires. The models were analyzed using Structural Equation Modeling. The results show that social support has a direct effect on mothers’ physical health and life satisfaction and an indirect effect, mediated through the occupational performance variables, on mothers’ mental health and life satisfaction. Role overload does not affect mothers’ health and life satisfaction. These results suggest that mothers could benefit from health programs that help them manage their occupational routines. Such programs should focus on improving the mother’s occupational performance and adapting her social environment to fit her occupational needs.

## 1. Introduction

One of the assumptions underlying theory and practice in the health professions in general and in occupational therapy in particular is the belief that there is a strong relationship between engagement in occupations, health, and wellbeing [1,2]. This assumption was tested in various studies, and although there is clear agreement about the existence of a relationship between the three variables, there is not enough evidence about the direction of the connection in different populations. In addition, other factors affect the relationship between the three variables. These factors, referred to as mediators, include stress, the degree of complexity of the occupation, and achieving a balance between various occupations [3].

**Figure 1:**
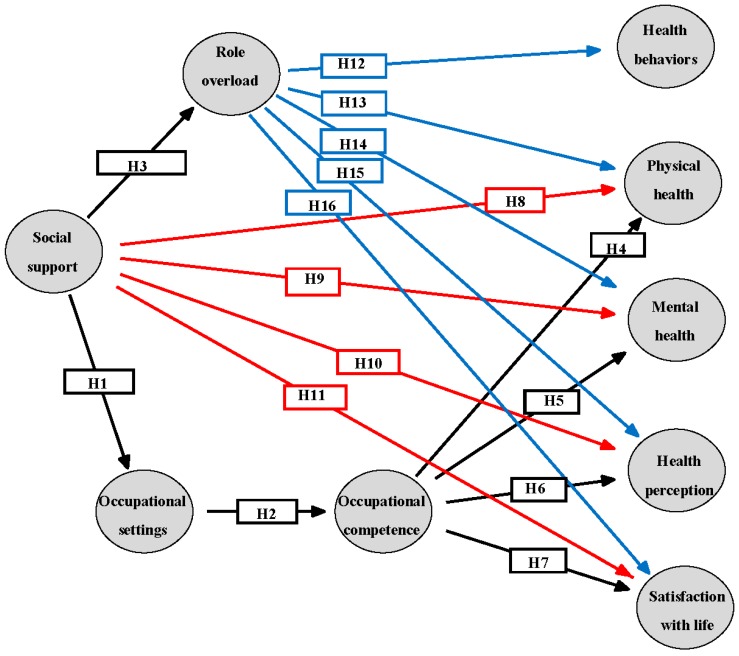
Three alternative theoretical models to describe mothers’ health and life satisfaction.

The following is a review of the literature regarding occupational performance, health, life satisfaction, related factors and their relationships, that establish the conceptual model for empirical testing. Subsequently, a proposed theoretical model has been outlined to illustrate the study’s hypotheses (Figure 1).

### 1.1. Occupational Performance (See Figure 1, Hypotheses 1 and 2)

Occupation encompasses all the activities that people do to occupy themselves, including looking after themselves, enjoying life, and contributing to the social and economic fabric of their communities [2]. The human capacity to choose occupations and perform them satisfactorily within the context of one’s life is referred to as occupational performance, which is the outcome of the dynamic relations between a person, the occupation, and the environment [4]. According to the Model of Human Occupation (MOHO) occupational performance is measured by three constructs: occupational identity, occupational competence, and occupational settings [5,6]. Occupational identity is a composite sense of who one is and wishes to become as an occupational being. Occupational competence refers to the ability to actualize a desired occupational identity in a way that provides satisfaction and meets environmental demands [5]. Occupational identity and competence are developed and realized together over time, as we develop and respond to life changes. Occupational settings refer to the everyday environment in which the person lives, carries out the productive role in one’s life, and engages in leisure activities. It is a composite of spaces and social groups that cohere and constitute a meaningful context for performance. Occupational settings affect occupational identity and competence. At the same time, occupational settings are greatly influenced by culture and social conditions [6]. When the person’s social surroundings at workplace, home and leisure sites are more supportive, his or her occupational settings have a better impact on the person’s occupational life [5,6].

### 1.2. Occupational Performance, Role Load, Health, and Life Satisfaction (See Figure 1, Hypotheses 3−7 and 12−16)

Participation in meaningful occupations promotes occupational performance and contributes to good health and wellbeing [1,7]. Lack of meaningful occupation or occupational role overload may have a negative effect on health and wellbeing [3]. Role overload occurs when individuals are subject to too many role demands and have too little time to meet them, which hinders their ability to address all the demands [8], resulting in frustration [9]. Tension and conflict can occur not only when individuals attempt to play multiple roles but also when a person lacks the necessary resources and support to meet the demands of a particular role [10].

Of particular interest to the present research is mothering, a role that is often associated with multiple demands. Women juggle roles such as paid work, household labor, and caring for children. These multiple demands may cause role overload, which can affect mothers’ physical and mental health as well as their life satisfaction [11,12].

### 1.3. Mothers’ Occupations, Role Load, and Health (See Figure 1, Hypotheses 12−16)

Mothering is one of the most central roles in a woman’s life, played by 81% of adult American women [13]. When asked to rate its importance, the majority of mothers considered mothering to be the most salient aspect in their lives [14]. Mothering is dynamic and changes over the lifespan. First-time mothers report that the routine changes in roles affect their personal wellbeing and health. The transition to mothering is a challenging process that demands a new way of organizing daily routines and a search for new occupational balance [15]. As children grow up, the mothering role shifts to being an invested participant in her children’s lives, such as providing financial and emotional support [16].

It has been found that when women become mothers, they abandon roles such as worker, volunteer, and hobbyist, and at the same time expand their role of caregiver. Moreover, mothers’ role load is significantly higher than that of non-mothers [17]. Mothers choose an increase in role load and a decrease in the number of roles in order to conform to social expectations about “good mothers.” This choice is most likely affected by perceptions about mothers being the main caregivers for the children [18]. Pearson [19] found that role overload in women’s life affects their immune system, causing physical or mental illness and a decrease in wellbeing. Role overload may also interfere with health-promoting behaviors such as balanced eating, rest and exercising, therefore might increase the risk of health problems [20]. Role balance has a buffering effect on the relationship between daily hassles (relationships with children, parents or in-laws, and other relatives) and health status [21].

### 1.4. Mothers’ Social Support and Health (See Figure 1, Hypotheses 8−11)

Another factor affecting women’s health and life satisfaction is social support [22,23]. There are several types of social support available to mothers (emotional and instrumental), and several sources for social support (family, friends, and professionals) [24]. For women who juggle multiple roles, having strong and positive support from others is correlated with a stronger immune system, higher life satisfaction, better health perception, and fewer symptoms of depression [25,26]. Relationship and marriage can promote women’s physical and mental health, but the quality of the relationship is critical, and one of the sources of tension in marriage is the sense of unfairness in the distribution of roles, *i.e.*, lack of support [23].

### 1.5. Mothers’ Socio-Demographic Factors and Health

Women’s health and life satisfaction are also positively related to socio-demographic factors such as years of education and socioeconomic status [27,28]. Other life circumstances having to do with employment status [27], the woman’s age [29], the number of children [30] and their ages [31] affect women’s health and should be taken into consideration when investigating women’s health and life satisfaction. Therefore, in the models investigated by the present study we controlled for education, hours at work, age of the mother, and the age and number of children.

In sum, women’s health and life satisfaction has been examined in relation to different variables such as role overload, sources of support, relationships, and different demographic variables. But no research has been conducted to explore how mother’s health and life satisfaction are related to occupational performance, taking into account role overload and social support and test these variables as a whole in a summarizing model. Therefore, the purpose of the present study was to examine three possible theoretical models to explain mothers’ health and life satisfaction from an occupational perspective (Figure 1). The relationships between the variables presented in the three models are based on the literature review and previous findings. According to the review occupational settings affect occupational competence [6], and occupational competence is related to health and life satisfaction [1,6,7]. Role overload is related to health and life satisfaction [11,12,19,20] and social support is both indirectly (mediated through occupational settings [5] and through role overload [21]), and directly related to health and life satisfaction [23,25,26], and therefore is an exogenous variable. Based on these previously reported relationships, the three models and the direction of the hypotheses were designed. Altogether, 16 hypotheses were tested, marked as H1-H16 in Figure 1. For example: Social support will have a positive effect on occupational settings (H1).

## 2. Materials and Methods

### 2.1. The Study Group

The study included 150 Israeli mothers. The inclusion criteria were: at least one child between the ages one to ten; age between 25–45 years; married; moderate socioeconomic status; between one and four children; at least 12 years of education; paid employment outside their home. In order to obtain a representative sample of the healthy population the exclusion criteria were mothers or children with an illness or disability (a known medical diagnosis). Participants were recruited by snowball sampling in the community.

### 2.2. Instruments

Data were collected by means of an interview and self-report questionnaires:

*The Occupational Performance History Interview—Second Version (OPHI-II)* is a semi-structured interview that explores the client’s occupational life history [5]. The interview is organized into five thematic areas: activity and occupational choices; critical life events; daily routines; occupational roles; and occupational settings. The information collected from these thematic areas was converted into three quantitative rating scales: occupational identity, occupational competence, and occupational settings. The three scales consist of a total of 29 items. Each item received a score between 1–4 points, with 4 representing exceptionally fitting occupational functioning and 1 representing extremely deficient occupational functioning. Assigning these ratings require clinical judgments. To make this process easier, criteria are written next to each item to assist the interviewer in assigning ratings. Interviews were graded separately for each scale, and then the ordinal variables were converted into interval ones according to “OPHI-II keys”, so that a score is obtained for each scale (for a total of three grades), ranging between 0–100 [5,6]. These scales were found to be valid across language and culture for an international sample of people with or without disabilities [32].

As a preliminary stage in this study, the OPHI-II was translated into Hebrew. Internal consistency reliability (Cronbach’s alpha) for the translated version was 0.87 for occupational identity scale, 0.82 for the occupational competence scale and 0.80 for occupational settings scale. Both occupational competence and occupational settings are represented in the study models as mediating variables. Occupational identity and occupational competence are highly correlated (*r* = 0.83, *p* < 0.001). As occupational competence incorporates occupational identity, it was chosen for the model over occupational identity.

*The modified Role Checklist (M-RCL)* is a self-administered measure based on the Role Checklist [33]. The M-RCL [17] includes two parts. The first is called “role loads list”, and the second is “role value” (identical to the original version). The “role loads list” has a load scale for each of the 10 roles (e.g., student, worker, care giver, *etc.*) in the past, present, or future. Besides indicating whether the participant had engaged in a certain role, he or she marks the load level experienced for each role for the three time periods on a scale of 1 (low occupational load) to 5 (very high occupational load). The average role load is then calculated for each time period. For the second component, “role value,” the participant is asked to mark the value each role holds, on a scale of 1 (not valued at all) to 3 (highly valued). The score is the sum for all 10 roles. Scores can range from 10–30. Higher scores indicate that the participant place higher importance/value to the listed roles (regardless of whether he/she is involved in this role). The M- RCL exhibits adequate test-retest reliability. Discriminant validity was found between mothers and non-mothers [17]. The average score of current roles for the ten occupational roles was entered as the mediating variable of role overload into the theoretical models.

*The Short Form Health Survey Questionnaire (SF-36)* is a well-established abbreviated survey designed to provide a multidimensional representation of health-related issues, from the participant’s perspective [34]. The questionnaire contains eight sub-scales based on 36 items, which are further grouped into two summarized categories: physical health and mental health, and an additional total health score [35]. The questionnaire is intended for various populations over the age of 14, both healthy and sick. The resulting score ranges from 0 to 100. A higher score indicates a better perception of one’s health state. The SF-36 was translated into Hebrew. The psychometric properties of the Hebrew version were good and resembled those reported by researchers in other countries [36]. Based on this instrument we entered two endogenous variables into the theoretical models: physical health and mental health, which were calculated based on the two summarized categories.

*The Self-Rated Health (SRH)* measures health perception. Is it a visual analogue scale (VAS) type of testing, which includes a single item, a 100-millimeter long straight horizontal line, marked 0 (the lowest) at one end and 10 (the highest) at the other. Participants mark a vertical line on the scale, which expresses their self-evaluation of their current state of general health [37,38]. The SRH can be used in epidemiological studies and health surveys among diverse populations [38]. The endogenous variable we entered into the theoretical models based on this instrument was health perception.

*The Satisfaction with Life Scale (SWLS)* evaluates life satisfaction in general. The SWLS assesses the cognitive component of subjective wellbeing (SWB). The questionnaire contains five statements, and participants are asked to rate their level of agreement on a 7-point scale [39]. All the items are summed to provide a final score ranging from 5 (minimum life satisfaction) to 35 (maximum life satisfaction) [40]. There is abundant support for the internal consistency and stability of the scale [41,42]. The SWLS was translated into Hebrew and was found as a valid and reliable scale that can be utilized in the Israeli context [41]. The endogenous variable we entered into the theoretical models based on this instrument was satisfaction with life.

*The Maternal Social Support Index (MSSI)* is an 18-item questionnaire designed to assess mother’s social support [43]. The MSSI is a standardized self-report measure consisting of scales that measure the mothers’ perception of daily task sharing among family members (11 items), satisfaction with relationships (three items), availability of emergency help and degree of community involvement (four items). Items are scored on different scales, ranging between 2 to 6 points. All the items are summed to provide one total score that can range from 1 to 39. Higher scores correspond to greater social support [44]. Construct validity has been established. Test-retest reliability was 0.75 [43]. The MSSI was translated into Hebrew for the purpose of this study. The exogenous variable we entered into the theoretical models based on this instrument was social support.

*The Health Behavior questionnaire* was developed for the present study and combines two previous questionnaires [45,46]. The purpose of the new health behavior questionnaire was to collect data on the behavior and state of health of the participants. The questionnaire contains questions about exercise habits, smoking habits, physical fitness, weight management, height, and medical history. According to the survey data on women’s health in Israel conducted by Ashkenazi and Gross [47], health behaviors include smoking, physical activity, and weight management. Based on this finding, the “health behaviors” variable in the present study was calculated based on the BMI, physical activity, and smoking variables. A BMI of 25 or higher was considered to reflect excess weight, thus indicating poor weight management [46]. A case in which a woman has never smoked is considered a healthy behavior [48,49], and a case in which a woman exercises three times a week or more is also considered a healthy behavior [50]. For each healthy behavior the participant received a score 1 and for each unhealthy behavior a score of 0. The final score ranged from 0 to 3. The endogenous variable we entered into the theoretical models based on this instrument was health behaviors.

### 2.3. Procedure

After participants gave their informed consent for inclusion in the study, two or three meetings were held with each one (depending on the length of the interview). The meetings took place in a quiet location that facilitated comfortable conversation (e.g., the participant’s home, workplace, coffee shop). The M-RCL was administered first to set the participant’s mind on her roles and occupations, followed by an hour and a half long interview based on the OPHI-II. All interviews began with explaining the term “occupation” to the interviewee in the following way: “Occupation is everything you do in your everyday life including: working, studying, taking care of others (childcare/parents), participating in volunteer activities, hobbies, social activities, home maintaining or religious activities”. If the interviewee focused on one or two occupations (*i.e*., thinking only of paid employment), then the interviewer asked again about the rest, reminding her of other occupations such as leisure, or childcare, making sure to ask about all the occupations in each interview. The remaining questionnaires were administered at the end of the interview in the following order: SF36, SRH, SWLS, MSSI, the Health Behavior questionnaire, and a demographic Questionnaire. Data collection was conducted by the first author and registered occupational therapists who were graduate students. All researchers learned to administer and score the OPHI-II by the first author. Both the occupational therapists and the first author scored at least two interviews individually and compared their rating to assure consistency of coding. The Behavioral Research Ethics Board of the University of Tel Aviv approved the project.

### 2.4. Data Analysis

Statistical measures (average, median, standard deviation, and range) were analyzed in order to describe each variable distribution. Pearson correlations were calculated between all the variables to describe their mutual distribution. These preliminary analyses, which provide vital information for the model validation phase, were performed using the SPSS 15.0 software (SPSS Inc., Chicago, IL, USA).

A confirmatory method of data analysis, Structural Equation Modeling (SEM) was used to test the three possible theoretical models on factors affecting mothers’ health and life satisfaction. This approach requires that relationships between variables be set a priori. This is an appropriate method of data analysis for the purpose of drawing conclusions [51]. SEM is based on a measurement and a structural model. The structural model describes the causal relationship between the latent variables, divided into exogenous and endogenous, with the former affecting the latter. The direction of the relationships was determined by the hypotheses [52].

The analysis was conducted using the EQS 6.1 software. Because of deviation from the normal distribution of study variables, maximum likelihood estimation was used to estimate the parameters, with robust correction [53], and using Satorra-Bentler χ^2^ (sb χ^2^) index. Because of the small sample size, the measurement part of the model was determined by fixing the lack of reliability of each latent variable using the formula (1 -α) × Ŝ^2^ to calculate measurement error variance (α and Ŝ^2^ represent Cronbach internal consistency and scale variance).

The validity of the models was tested by comparing three alternative nested models [54]. All three models include the effect of social support on role overload and occupational settings, and the effect of occupational settings on occupational competence. The differences between the models are based on the different effects on the variables of health and life satisfaction, as found in the literature. Model I (Figure 1, arrows in black), the simplest model tested, includes the effects of occupational competence on life satisfaction and on the health variables (except for health behavior). Model II (Figure 1, arrows in black and dotted red), elaborates on the previous model and includes also the direct effects of social support on life satisfaction and on the health variables (except for health behavior). Model III (Figure 1, adding the blue arrows), the most expanded model, also includes the effects of role overload on all health variables and on life satisfaction. This method allows assessing the degree of sufficiency and parsimony of the models before testing their plausibility—three attributes necessary for the appropriate model. Before the confirmatory stage of the statistical analysis and SEM, an exploratory stage was conducted. The additional parameters included in the three models are correlations between control variables (mother’s age, education, number of children, age of children, and working hours) and correlations between health variables and life satisfaction.

Because three attributes are necessary for an appropriate model (sufficiency, parsimony, and plausibility), three types of goodness of fit were chosen to evaluate and compare the tested models: absolute fit indices, incremental fit indices, and parsimony fit indices [55,56]. Absolute goodness of fit was estimated by the Goodness of Fit Index (GFI), χ^2^, and by the Standardized Root Mean Squared Residual (SRMSR). The indices of fit (null model) were the Comparative Fit Index (CFI) and the Normed Fit Index (NFI). The Adjusted Goodness of Fit Index (AGFI) and the Parsimonious Normed Fit Index (PNFI) were used as parsimony fit indices to correct the incremental fit indices for non-efficiency with respect to the relationship between the degrees of freedom of the model tested and the degrees of freedom of the null model.

Low and non-significant χ^2^, with a value higher than 0.90 for the GFI, CFI, and NFI, and lower than 0.10 for the SRMSR were considered to be an indication of acceptable goodness of fit. The comparison of alternative models was conducted with respect to each of the indices. Statistical tests were conducted only for χ^2^.

## 3. Results

### 3.1. Sample Characteristics

All 150 mothers were married women, with a mean age of 34.25 (SD = 4.78), living in the same house with their husbands and children. The average age of all children was 5 years (SD = 3.66). Participants had a mean of 15.71 (SD = 2.23) years of education (Table 1). Participants worked in paid employment outside their home on average 34.89 h per week (SD = 9.82). They reported spending an average of 46.15 h per week (SD = 13.27) caring for their children and 8.84 h per week (SD = 7.04) engaging in leisure activities. Most participants were born in Israel (90%) and were secular (80%). Based on a residential neighborhood, the sample included women from moderate to high socio-economic status (based on the Israeli Central Bureau of Statistics [57] living area clusters).

Participants in this study reported not having significant physical or mental problems at the present or in the past. The sample was characterized by a low rate of health conditions such as blood lipids (*n* = 4), blood pressure (*n* = 6), cholesterol (*n* = 12), and asthma (*n* = 3). Over the past year they had seen a physician only 1.84 times (SD = 2.12), and they did not undergo hospitalization or surgery.

**Table 1:** Demographic variables of the study population: Nominal variables (N = 150).

Variable Name	Levels	Frequency	Percentage
No. of children	1	41	27.3
	2	67	44.7
	3	35	23.3
	4	7	4.7
Education	High school	24	16
	Bachelor’s degree	59	39.3
	Two Bachelor’s degrees	6	4
	MA	30	20
	PhD	5	3.3
	Other degrees	26	17.3

### 3.2. Distribution of Study Variables

The means, medians, standard deviations, ranges, skewness, and kurtosis of all the variables included in the model and background variables are presented in Table 2. These descriptive findings, relating to the distribution of the study variables, led to the decision to use robust correction to estimate the maximum-likelihood method in the analysis of the structural equations [53].

**Table 2:** Descriptive statistics of study variables (N = 150).

Variable	Mean	Median	SD	Range (Min−Max)	Skewness	Kurtosis
1. Social support	27.02	27.00	4.29	(15–36)	−0.21	−0.31
2. Occupational settings	67.00	67.00	11.19	(39–91)	−0.21	−0.48
3. Occupational competence	69.03	68.00	10.80	(44–100)	0.26	−0.34
4. Role overload	2.35	2.40	0.41	(1.40–3.50)	−0.12	−0.31
5. Health behaviors	1.69	2.00	0.70	(0–3)	−0.31	0.06
6. Physical health	76.83	79.00	14.19	(19–99)	−1.25	1.94
7. Mental health	76.07	78.50	12.93	(37–98)	−1.14	1.16
8. Health perception	84.07	88.00	14.33	(35–100)	−1.19	1.11
9. Satisfaction with life	26.93	28.00	4.70	(10–34)	−1.17	1.59
10. Mother’s age	34.25	33.00	4.78	(25–45)	0.32	−0.52
11. Education	15.71	16.00	2.23	(12–23)	0.03	0.07
12. No. children	2.05	2.00	0.83	(1–4)	0.39	−0.47
13. Average children’s age	5.00	3.71	3.66	(1–15.75)	1.04	0.10
14. Hours at work	34.89	35.00	9.82	(12–62)	−0.02	−0.06

### 3.3. Correlations between Research Variables

The simple correlations between research variables shown in Table 3 (under the main diagonal line) show that some are compatible with the research hypothesis and others are not.

**Table 3:** Pearson correlations (simple correlations below and correction for attenuation above the main diagonal line) between all study variables (N = 150).

Variable	1	2	3	4	5	6	7	8	9	10	11	12	13	14
1. Social support	(0.63)	0.24	0.29	0.39	0.10	0.21	0.14	0.11	0.50	0.03	0.16	0.16	0.06	0.05
2. Occupational settings	0.17 *	(0.80)	0.85	0.10	0.21	0.25	0.42	0.04	0.42	0.30	0.04	0.36	0.38	−0.06
3. Occupational competence	0.21 *	0.69 ***	(0.82)	0.07	0.21	0.08	0.20	0.04	0.43	0.23	0.20	0.33	0.29	−0.03
4. Role overload	0.31 ***	0.09	0.06	-----	0.13	0.18	0.05	0.17	0.18	−0.13	0.12	−0.09	−0.10	0.14
5. Health behaviors	0.08	0.19 *	0.19 *	0.13	-----	0.24	0.13	0.15	0.17	−0.10	0.06	0.02	0.02	0.13
6. Physical health	0.14	0.19 *	0.06	0.15	0.20 *	(0.70)	0.77	0.66	0.34	0.06	−0.13	0.14	0.14	0.07
7. Mental health	0.09	0.31 ***	0.15	0.04	0.11	0.53 ***	(0.67)	0.54	0.69	0.09	−0.16	0.15	0.12	−0.22
8. Health perception	0.09	0.04	0.04	0.17 *	0.15	0.55 ***	0.44 ***	-----	0.25	−0.19	−0.05	−0.03	−0.10	0.07
9. Satisfaction with life	0.36 ***	0.34 ***	0.35 ***	0.16	0.15	0.26 ***	0.51 ***	0.23 **	(0.82)	0	0.08	0.13	0.06	−0.12
10. Mother’s age	0.02	0.27 ***	0.21 *	−0.13	−0.10	0.05	0.07	−0.19 *	0	-----	0.13	0.54	0.77	−0.07
11. Education	0.13	0.04	0.18 *	0.12	0.06	−0.11	−0.13	−0.05	0.07	0.13	-----	−0.08	0.01	0.03
12. No. children	0.13	0.32 ***	0.30 ***	−0.09	0.02	0.12	0.12	−0.03	0.12	0.54 ***	−0.08	-----	0.63	−0.07
13. Children age average	0.05	0.34 ***	0.26 **	−0.10	0.02	0.12	0.10	−0.10	0.05	0.77 ***	0.01	0.63 ***	-----	−0.08
14. Hours at work	0.04	−0.05	−0.03	0.14	0.13	0.06	−0.18 *	0.07	−0.11	−0.07	0.03	−0.07	−0.08	-----

*Notes*: * *p* < 0.05, ** *p* < 0.01, *** *p* < 0.005. Values in bold along the main diagonal line represent reliability and internal consistency (Cronbach’s alpha). For the role overload, health behaviors, health perception, and background variables Cronbach’s alpha values were not calculated (some are objective background variables that should not contain measurement error, and for some it was not possible to calculate reliability). The simple correlations between research variables are under the main diagonal line. Correction for attenuation (above the main diagonal line) was performed using the formula $r_{xy\;\left(corrected\right)}=\frac{r_{xy\;\left(observed\right)}}{\sqrt{\alpha_{x}}\times \sqrt{\alpha_{y}}}$ [58].

The weak reliability (internal consistency on the main diagonal line) reduces the statistical correlation and power. Correction for attenuation, as performed in SEM, increases the strength of correlations, as we can see above the main diagonal line. For example, the positive correlation between social support and occupational settings (*r* = 0.17 before correction for attenuation) is stronger after the correction (*r* = 0.24). The corrected correlations are used in the SEM.

### 3.4. Testing of the Theoretical Models

Testing of the theoretical models was accomplished by comparing the goodness of fit of the three alternative nested models [54] (Table 4). The results show that Model I has acceptable goodness of fit only for some of the fit indices. χ^2^ was significant, indicating a significant deviation between the matrix of the observed covariances and the matrix of the covariances reconstructed by the model. Moreover, NFI was somewhat lower than needed. The rest of the fit indices were within acceptable range.

**Table 4:** The goodness of fit of the three alternative nested models (N = 150).

Model	s-b χ^2^	df	GFI	SRMSR	CFI ^a^	NFI ^a^	AGFI	PNFI ^b^
Model I: Occupational competence effects on health and life satisfaction	39.63 **	19	0.964	0.051	0.909	0.881	0.801	0.159
Model II: Model I + the direct effects of social support	20.84	15	0.980	0.035	0.974	0.937	0.862	0.134
Model III: Model II + the effects of role overload	17.21	10	0.984	0.030	0.968	0.948	0.828	0.090

Notes: * *p* < 0.05, ** *p* < 0.01, *** *p* < 0.005. The index value of chi-square for the null model does not explain at all the matrix of covariances: χ^2^(105) = 331.95. **^a^** Robust index, calculated using s-b χ^2^; **^b^** Parsimony index calculated using the parsimony ratio between the degrees of freedom of the tested model to the degrees of freedom of the null model. A value higher than 0.90 for the GFI, CFI and NFI indices and lower than 0.10 for the SRMSR index is considered an indication of acceptable goodness of fit.

**Figure 2:**
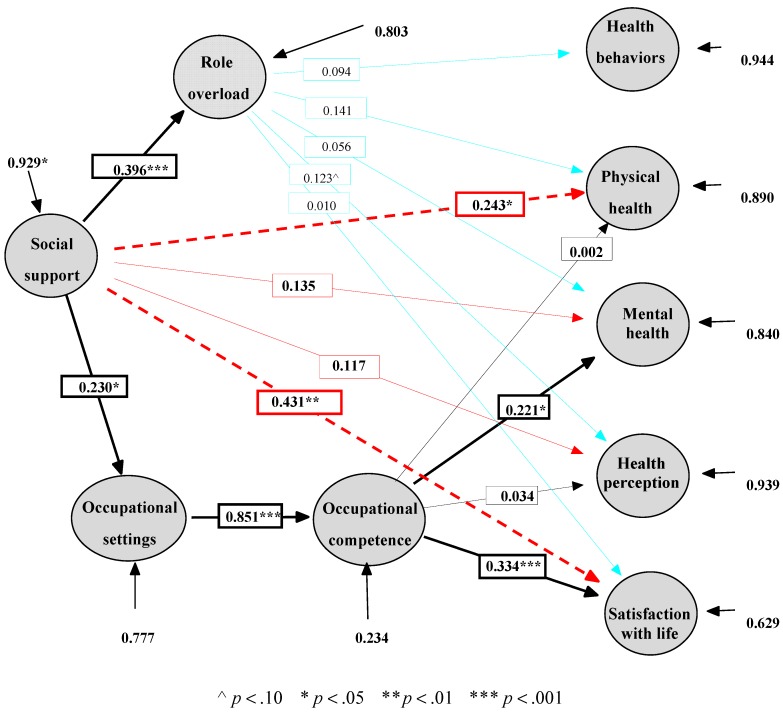
Parameter estimates in the three models (N = 150). Figure 2 shows standards estimates. The value at the beginning of an external line indicates standard error variance (percentage of unexplained variance in the endogenous variable). The bright line indicates that the effect is not significant. Black lines describe Model I, dotted red lines describe the additional effects in Model II, and blue lines describe the additional effects in Model III.

By contrast, the data did not reject Model II in any way, and it can be considered a plausible model. Furthermore, Model II has a clear priority over the simpler Model I (Δs-b χ^2^(4) = 18.79, *p* < 0.001) and therefore makes a greater contribution to explaining the correlations between the research variables. Model II fits the data in a manner similar to expanded Model III, and it is therefore not disadvantaged relative to Model III (Δs-b χ^2^(5) = 3.63, *p* > 0.05, ΔGFI = −0.004, ΔSRMSR = 0.005, ΔCFI = 0.006, ΔNFI = −0.011). Model II has also been found to be more parsimonious than Model III regarding to the number of degrees of freedom (ΔAGFI = 0.034, ΔPNFI = 0.044). Therefore, the results show that Model II is the most plausible, sufficient, and parsimonious of the three models tested. Figure 2 presents the parameter estimates in the three models.

Five out of seven estimated effects (Figure 2) were significant in Model I (black), two out of four additional effects were significant in Model II (dotted red), and none of the other effects in Model III (blue line) was found to be significant. Altogether, seven hypotheses out of 11 (Figure 1) were confirmed by Model II. For example, social support positively affects physical health and therefore H8 was confirmed.

The direct significant effects found between social support and two of the health and satisfaction variables (physical health (H8) and life satisfaction (H11)) indicate that the mediation of the effect of social support on health and life satisfaction is only partial. In other words, social support has a positive effect on health variables and life satisfaction, both directly (the effect on physical health and life satisfaction) and indirectly, mediated through its effect on occupational performance variables as follows: social support has a positive effect on occupational settings (H1), which positively affect occupational competence (H2), which in turn has a positive effect on mother’s mental health (H5) and on life satisfaction (H7).

Role overload, however, does not affect mothers’ health and life satisfaction (H12−H16 were rejected). Therefore, it cannot be claimed that some of the effects of social support on health and life satisfaction are also mediated by role overload, despite the finding that social support has a positive effect on role overload (H3).

The explained variance in the endogenous variables in the selected model was low for most health variables (5.6% from the variance of health behaviors, 11% from the variance of physical health, 16% from the variance of mental health, and 6.1% from the variance of health perception), except for life satisfaction (37.1%). A low percentage of explained variance was also shown in role load (about 20%) and occupational settings (23.3%). Only the variance in occupational competence is explained to a considerable degree (76.6%). Thus, the existing variables in the model, including the control variables, most likely cannot provide the only explanation for the variance of the explained variables (the values of standard error variance are shown in Figure 2).

## 4. Discussion

The present study explores further the relationship between occupation, health, and wellbeing and factors mediating this relationship among a population of mothers. Based on the literature review and on previous findings, three possible theoretical models were constructed to explain mothers’ health and life satisfaction. The results show that the second model of the three has the best fit, and it is therefore the most plausible, sufficient, and parsimonious of the three models tested. According to this model, social support has a direct effect on mothers’ physical health and life satisfaction and an indirect effect, mediated by the occupational performance variables, on mothers’ mental health and life satisfaction.

### 4.1. The Effect of Occupational Settings and Occupational Competence on Health and Life Satisfaction

It was found that occupational performance scales affect health and life satisfaction. This finding reinforces the assumption that there is a strong relationship between occupation, health, and wellbeing [3]. Consistent with Kielhofner [6], the results of the present study demonstrate that when interaction with others are supportive and create a good social atmosphere (*i.e.*, a competent occupational setting), occupational competence improves. This finding also supports many theoretical models maintaining that the environment affects participation and occupational performance [4,7]. The novelty of this study is focusing on mothers and demonstrating the importance of adapting mother’s occupational setting to fit her occupational needs, which will eventually affect the way she functions in her everyday occupations.

The results also highlight the way in which health and life satisfaction are affected by occupational competence. Results indicated that higher occupational competence enhances mental health among mothers. In previous studies mothers reported mental stress as a result of the intensive care their children require [16,59,60], and lack of time for themselves [61]. In particular, mothers attach greater meaning and importance to mental health than they do to their physical health [62]. In a study by Lewis and Ridge [61], mothers attributed the benefits of their mental rather than physical health to participating in leisure activities, such as exercise classes. They argued that mental health or “their sanity” is what enables them to continue to care for their children and be good mothers. At the same time, our results indicated that occupational competence does not affect mothers’ physical health. Previous studies have reported a relationship between mothers’ participation in parental occupations and the presence of physical health problems such as musculoskeletal impairments [31,63]. While taking care of their children, mothers often use harmful lifting techniques. Mothers are also engaged in other tasks such as preparing meals, shopping for groceries, and cleaning, often in parallel with playing with the children [64]. The nature of these tasks causes musculoskeletal problems [59]. Several factors may have contributed to constructing the mothers’ occupational competence and neutralized its effect on physical health in the current study: the fact that mothers do not “allow themselves” to be physically sick, and therefore consciously or unconsciously do not allow their occupational functioning to affect their physical health; the young age of the participants in the present study (before the time when chronic conditions typically develop) [29]; and the fact that the women spent many hours in leisure activities a factor that protects their physical health [31].

According to the present study, occupational competence also has a positive effect on the mothers’ life satisfaction. Daily occupations in which the family engages affect the parents’ sense of competence and their life satisfaction [65], particularly that of the mothers [66]. Competence is an essential foundation for wellbeing [67]. According to a study by Reis *et al.* [68], competence positively affects wellbeing. They defined competence as the extent to which people can effectively bring about the outcomes they desire in their activities, a definition similar to that of occupational competence used in the present study.

In sum, to date mothers’ health was examined in relation to many variables, e.g., demographic variables [30], personality traits [69], and variables related to the woman’s environment [26] or genetics [70]. The current study shows that these factors do not paint a complete picture. The effect of occupational performance on mothers’ health and life satisfaction is a new finding that illuminates the importance of a sense of competence in woman’s life. This finding reinforces the importance of the mothers’ ability to pursue their occupations and highlights the health risk that arises when occupational competence declines. Since occupational competence includes “participating in a range of occupations…” ([6], p. 107) and high scores in the occupational competence scale reflects involvement in variety of roles, the results of this study might indicate that mothers who do not engage in a wide range of occupations compromise their mental health and reduce their life satisfaction. The effect on their children has yet to be studied.

### 4.2. The Effect of Social Support on Health and Life Satisfaction

The findings of the present study indicate that social support has a positive effect on health and life satisfaction. Thus, improving the mothers’ social support leads to enhanced physical and mental health and improved life satisfaction. Social support is a vital resource for maintaining health and wellbeing [7,24,71]. Previous studies [23,64] have already pointed out the effect of social support on physical health, in particular the effect of instrumental support (e.g., obtaining help in various household tasks). Receiving help in everyday tasks contributes to the mothers’ physical health because it reduces musculoskeletal exertion when caring for toddlers and dealing with households tasks [64]. Therefore, social support has a direct effect on mothers’ physical health, but its effect on mothers’ mental health is not direct, and it is mediated by occupational performance variables. Adequate social support can make a working place, home, or place of entertainment a better environment for the person, improving one’s occupational settings [5]. Improved occupational settings in turn improve occupational competence and increase mothers’ mental health.

Mothers’ health perception was also measured in relation to social support, and it was found that increased social support does not lead to higher health perception, contrary to previous findings. A few studies found a positive correlation between social support, as expressed in social networks or a sense of belonging, and health perception [62,72]. The wide distribution of the health perception variable may be responsible for the lack of effect of social support on it. A study by Knesebeck and Geyer [73] examined the relationship between social support and health perception in women from 22 European countries. The findings show a correlation between the variables in only 50% of the countries.

In sum, social support is of great importance in mothers’ lives; it enables them to function in their everyday occupations and it benefits their health and satisfaction with life.

### 4.3. The Effect of Role Overload on Health and Life Satisfaction

The findings of this study show that greater social support increases role overload. Although initially this seems counterintuitive, social support allows participation in activities [7], and as mothers have more support from their social environment they allow themselves to become involved in more occupational roles which increases their load. This finding differs from those of most previous studies investigating the association between social support and role overload. Previous findings indicate that support reduces the load by making it easier for the mothers and diminishing their load [74]. But Doyal [75] argued that an extensive social network can be a disadvantage rather than an advantage because it encourages women to increase their participation in different occupations, and therefore increases the “double burden” resulting from the multitude of occupational roles.

Our findings suggest, however, that role overload does not affect mothers’ health and life satisfaction, and therefore what affects health and life satisfaction is the ability to perform the occupations (occupational competence) and not the overload. According to our findings, provided that women enjoy adequate social support, they can take on many occupations without fear for their health because their health is not affected by overload, and what ultimately determines their health and life satisfaction is how they perform these occupations.

Rejection of the hypotheses concerning the effect of social support on health mediated by role overload raises a question about the way in which role overload was measured and understood in the present study. Understanding the concept of load was up to each participant’s interpretation of each of the roles she performed. Role overload occurs when an individual has too many role demands and too little time to fulfill them [8] but the dimension of time was not reflected in the M-RCL instructions given to participants.

Recent studies have emphasized the subjective experience rather than the actual circumstances [76,77]. These studies found that it is the significance of the roles for the women, or the values of these roles that determine women’s health, rather than the number of the roles. The meaning ascribed to occupational roles has been discussed previously [78,79], but it has not been tested as a variable in the models of this study. This may indicate that “meaning” is another mediating variable that appears between role overload and health.

### 4.4. Limitations and Recommendations for Future Studies

The present study was limited in its representativeness. Only married, educated, middle-class women who had no illness or disability, and who had no children with illness or disability were targeted. Therefore, the findings cannot be generalized to other groups of women. Further studies among mothers from various cultures and different socio-demographic backgrounds are needed. The study used research tools that rely on self-report. Although all the tools used have acceptable reliability and validity, they all measure variables from the perspective of the mother and are affected by her personality, disposition, or the events that occurred close to the time when the questionnaires were completed. Future studies testing the extended model should consider measuring the concept of role load while focusing on subjective meaning ascribed to the array of roles and incorporating the role value in the role load score.

## 5. Conclusions

To date, motherhood has been scarcely examined from the perspective of occupation and its effect on the health and life satisfaction of women. The results of the present study amount to a new theoretical model that explains mothers’ health and life satisfaction from an occupational perspective. Our results indicate that occupational performance affects mental health and life satisfaction. In this sense, it is a trailblazing study in the field of occupational therapy, and among the first studies to have found empirical evidence of this effect. The research also indicates that social support has a direct effect on physical health and life satisfaction and an indirect effect on mental health. Role overload does not mediate the effect of social support on health and life satisfaction, indicating that it is the women’s ability to perform their occupations and not their load that affects their health and life satisfaction. The study suggests that family-centered intervention should include close attention to the mothers’ support needs, using an occupational lens, strengthening mothers’ occupational competence and emphasizing the importance of participation in a variety of occupations that meet the mother’s interests and are compatible with her occupational identity. Continued exploration of this occupation is essential to improving family-centered practice.

## References

[1] Hocking C., Crepeau E.B., Cohn E.S., Schell B.A.B. (2009). Contribution of occupation to health and well-being. Willard & Spackman’s Occupational Therapy.

[2] Townsend E.A., Polatajko H. (2013). Enabling Occupation II: Advancing an Occupational Therapy Vision for Health, Well-Being, and Justice through Occupation.

[3] Law M., Steinwender S., Leclair L. (1998). Occupation, health and well-being. Can. J. Occup. Ther..

[4] Law M., Cooper B., Strong S., Stewart D., Rigby P., Letts L. (1996). Person-environment-occupation model: A transactive approach to occupational performance. Can. J. Occup. Ther..

[5] Kielhofner G., Mallinson T., Crawford C., Nowak M., Rigby M., Henry A., Walens D. (2004). A User’s Manual for the Occupational Performance History Interview— Version 2.1 OPHI-II.

[6] Kielhofner G. (2008). Model of Human Occupation: Theory and Application.

[7] Baum M.C., Christiansen C.H., Christiansen C.H., Baum M.C., Haugen B. (2005). Person-environment-occupation-performance: An occupation-based framework for practice. Occupational Therapy: Performance, Participation, and Well-Being.

[8] Coverman S. (1989). Role overload, role conflict, and stress: Addressing the consequences of multiple role demands. Soc. Forces.

[9] Booth A., Johnson D.R., Granger D.A. (2005). Testosterone, marital quality, and role overload. J. Marriage Fam..

[10] Goldberg W.A., Greenberger E., Hamill S., O’Neil R. (1992). Role demands in the lives of employed single mothers with preschoolers. J. Fam. Issues.

[11] Glynn K., Maclean H., Forte T., Cohen M. (2009). The Association between role overload and women’s mental health. J. Womens Health.

[12] Kane P. Women and Occupational Health, World Health Organization, Geneva, Switzerland. http://apps.who.int/iris/handle/10665/65855?mode=full&submit_simple=Show+full+item+record.

[13] U.S. Census Bureau (2010). Fertility of American Women: 2010—Detailed Tables.

[14] Avrech Bar M., Labock-Gal D., Jarus T. (2011). Occupational performance, social support and life satisfaction in single mothers compared with married mothers. Isr. J. Occup. Ther..

[15] Hunter H.M., Blair S.E.E. (2000). The magic, mess and muddles of becoming a mother—An occupational perspective. Occupation.

[16] Francis-Connolly E. (2000). Toward understanding of mothering: A comparison of two motherhood stages. Am. J. Occup. Ther..

[17] Avrech Bar M., Rubin V., Gavrieal-Tyjchman G., Jarus T. (2013). The validity and reliability of the modified version of the Role Checklist (M-RCL). Scand. J. Occup. Ther..

[18] Koniak-Griffin D., Logsdon M.C., Hines-Martin V., Turner C.C. (2006). Contemporary mothering in a diverse society. JOGNN.

[19] Pearson Q.M. (2008). Role overload, job satisfaction, leisure satisfaction, and psychological health among employed women. J. Counsel. Dev..

[20] Doumas D., Margolin G., John R.S. (2003). The relationship between daily marital interaction, work and health-promoting behaviours in dual earner couples: An extension of the work-family spillover model. J. Fam. Issues.

[21] Stuart G., Garrison M. (2002). The influence of daily hassles and role balance on health status: A study of mothers of grade school children. Women Health.

[22] Leahy-Warren P., McCarthy G., Corcoran P. (2012). First-time mothers: Social support, maternal parental self-efficacy and postnatal depression. J. Clin. Nurs..

[23] Williams K., Umberson D., Goldman M.B., Hatch M.C. (2000). Women, stress, and health. Women and Health.

[24] Börjesson B., Paperin C., Lindel M. (2004). Maternal support during the first year of infancy. J. Adv. Nurs..

[25] Arborelius E.U., Bremberg S.G. (2003). Supportive and nonsupportive qualities of child health nurses’ contacts with strained infant mothers. Scand. J. Caring Sci..

[26] Tardy R.W. (2000). But I am a good mom: The social construction of motherhood through health-care conversations. J. Contemp. Ethnogr..

[27] McDonough P., Walters V., Strohschein L. (2002). Chronic stress and the social patterning of women’s health in Canada. Soc. Sci. Med..

[28] Rowland Hogue C.J., Goldman M.B., Hatch M.C. (2000). Gender, race, and class: From epidemiologic association to etiologic hypotheses. Women and Health.

[29] Goldman M.B., Hatch M.C., Goldman M.B., Hatch M.C. (2000). An overview of women and health. Women and Health.

[30] Matthews S., Power C. (2002). Socio-economic gradients in psychological distress: A focus on women, social roles and work-home characteristics. Soc. Sci. Med..

[31] Sanders M.J., Morse T. (2005). The ergonomics of caring for children: An exploratory study. Am. J. Occup. Ther..

[32] Kielhofner G., Mallinson T., Forsyth K., Lai J.-S. (2001). Psychometric properties of the second version of the Occupational Performance History Interview (OPHI-II). Am. J. Occup. Ther..

[33] Oakley F. (1982). The Model of Human Occupation in Psychiatry.

[34] Ware J.E., Sherbourne C.D. (1992). The MOS 36-Item Short-Form health survey (SF-36): I, Conceptual framework and item selection. Med. Care.

[35] Kalantar-Zadeh K., Kopple J.D., Block G., Humphreys M.H. (2001). Association among SF36 quality of life measures and nutrition, hospitalization, and mortality in hemodialysis. J. Am. Soc. Nephrol..

[36] Lewin-Epstein N., Sagiv-Schifter T., Shabtal E.L., Shmueli A. (1998). Validation of the 36-item short-form health survey (Hebrew version) in the adult population of Israel. Med. Care.

[37] Paul-Dauphin A., Guillemin F., Virion J.M., Briancon S. (1999). Bias and precision in visual analogue scales: A randomized controlled trial. Am. J. Epidemiol..

[38] Miilunpalo S., Vuori I., Oja P., Pasanen M., Urponen H. (1997). Self-rated health status as a health measure: The predictive value of self-reported health status on the use of physician services and on mortality in the working-age population. J. Clin. Epidemiol..

[39] Diener E., Emmons R.A., Larsen R.J., Griffin S. (1985). The satisfaction with life scale. J. Pers. Assess..

[40] Pavot W., Diener E. (1993). Review of the satisfaction with life scale. Psychol. Assess..

[41] Anaby D., Jarus T., Zumbo D.B. (2010). Psychometric evaluation of the Hebrew language version of the satisfaction with life scale. Soc. Indic. Res..

[42] Vassar M. (2008). A note on the score reliability for the satisfaction with life scale: An RG study. Soc. Indic. Res..

[43] Pascoe J.M., French J. (1990). The reliability and validity of the Maternal Social Support Index for primiparous mothers: A brief report. Fam. Med..

[44] McCurdy K. (2001). Can home visitation enhance maternal social support?. Am. J. Community Psychol..

[45] Froom P., Melamed S., Triber I., Ratson N.Z., Hermoni D. (2004). Predicting self-reported health: The CORDIS study. Prev. Med..

[46] Center for Disease Control (2003). Mabat Survey of Health Condition and Nutrition.

[47] Ashkenazi Y., Gross R. (2004). Health Condition of Women in Israel: Review of Data Women’s Health in Israel.

[48] Mi-Ami N. A Background Document on Addiction to Smoking Cigarettes.

[49] Husten C.G., Malarcher A.M., Goldman M.B., Hatch M.C. (2000). Cigarette smoking: Trends, determinants, and health effects. Women and Health.

[50] Brezis M. Position Paper and Recommendations on Promoting Physical Activity in Israel. http://www.hadassah.org.il/media/1920578/______________________________.pdf.

[51] Dion P.A. (2008). Interpreting Structural Equation Modeling results: A reply to Martin and Cullen. J. Bus. Ethics.

[52] Bollen K.A. (1989). Structural Equations with Latent Variables.

[53] Bentler P.M. (1995). EQS: Structural Equation Program Manual.

[54] Anderson J.C., Gerbing D.W. (1988). Structural equation modeling in practice: A review and recommended two-step approach. Psychol. Bull..

[55] Boomsma A. (2000). Reporting analyses of covariance structures. Struct. Equ. Model..

[56] Hair J.F., Anderson R.E., Tatham R.L., Black W.C. (2006). Multivariate Data Analysis with Readings.

[57] Central Bureau of Statistics Living Area Clusters by Socio-Economic Level of the Population. http://www.cbs.gov.il/mifkad/hesber/mavo13.doc.

[58] Bedian A.G., Day D.V., Kelloway K.E. (1997). Correcting for measurement error attenuation in structural equation models: Some important reminders. Educ. Psychol. Meas..

[59] Griffin S.D., Price V.J. (2000). Living with lifting: Mothers’ perceptions of lifting and back strain in childcare. Occup. Ther. Int..

[60] Olson J.A., Esdaile S.A., Olson J.A. (2004). Mothering co-occupations in caring for infants and young children. Mothering Occupations: Challenge, Agency and Participation.

[61] Lewis B., Ridge D. (2005). Mothers reframing physical activity: Family oriented politicism, transgression and contested expertise in Australia. Soc. Sci. Med..

[62] Hale C.J., Hannum J.W., Espelage D.L. (2005). Social support and physical health: The importance of belonging. J. Am. Coll. Health.

[63] Vincent R., Hocking C. (2013). Factors that might give rise to musculoskeletal disorders when mothers lift children in the home. Physiother. Res. Int..

[64] Primeau L. (2000). Divisions of household work, routines, and child care occupations in families. J. Occup. Sci..

[65] Churchill S., Stoneman Z. (2004). Correlates of family routines in Head Start Families. Early Child Res. Pract..

[66] Evans J., Rodger S. (2008). Mealtimes and bedtimes: Window to family routines and rituals. J. Occup. Sci..

[67] Deci E.L., Ryan R.M. (2000). The “what” and “why” of goal pursuits. Human needs and the self determination of behavior. Psychol. Inq..

[68] Reis H.T., Sheldon K.M., Gable S.L., Roscoe J., Ryan R.M. (2000). Daily well-being: The role of autonomy, competence, and relatedness. Pers. Soc. Psychol. B..

[69] Kenney J.W., Bhattacharjee A. (2000). Interactive model of women’s stressors, personality traits and health problems. J. Adv. Nurs..

[70] Wilkins-Haug L., Erickson K., Hill L., Power M., Holzman G.B., Schulkin J. (2000). Obstetrician-gynecologists’ opinions and attitudes on the role of genetics in women’s health. J. Women Health Gen. B.

[71] Wang H.H., Wu S.Z., Liu Y.Y. (2003). Association between social support and health outcomes: A meta-analysis. Kaohsiung J. Med. Sci..

[72] Hyyppä M.T., Mäki J. (2001). Individual-level relationships between social capital and self-rated health in a bilingual community. Prev. Med..

[73] Knesebeck O.V.D., Geyer S. (2007). Emotional support, education and self-rated health in 22 European countries. BMC Public Health.

[74] Gevir D., Goldstand S., Weintraub N., Parush S. (2006). A comparison of time use between mothers of children with and without disabilities. OTJR.

[75] Doyal L. (1995). What Makes Women Sick? Gender and Political Economy of Health.

[76] Anaby D., Jarus T., Backman C.L., Zumbo B.D. (2010). The role of occupational characteristics and occupational imbalance in explaining well-being. Appl. Res. Qual. Life.

[77] McMunn A., Bartley M., Kuh D. (2006). Women’s health in mid-life: Life course social roles and agency as quality. Soc. Sci. Med..

[78] Hammell K.W. (2004). Dimensions of meaning in the occupations of daily life. Can. J. Occup. Ther..

[79] Reed K., Hocking C., Smythe L. (2010). The interconnected meanings of occupation: The call, being-with, possibilities. J. Occup. Sci..

